# Genome Sequences of *Apilactobacillus kunkeei* Strains, Mannitol-Producing Bacteria Isolated from Nectariferous Plants

**DOI:** 10.1128/mra.00892-21

**Published:** 2022-01-20

**Authors:** Klaudia Gustaw, Piotr Koper, Adam Waśko

**Affiliations:** a Department of Biotechnology, Microbiology, and Human Nutrition, Faculty of Food Science and Biotechnology, University of Life Sciences in Lublin, Lublin, Poland; b Department of Genetics and Microbiology, Institute of Biological Sciences, Maria Curie-Skłodowska University, Lublin, Poland; University of Arizona

## Abstract

In this announcement, we report the genome sequences of two newly isolated Apilactobacillus kunkeei strains, strain 7K11C and strain 7K4AA, which are plasmid harboring and were isolated from flowers in wastelands in Poland. Furthermore, we present the longest sequence of the A. kunkeei DSMZ 12361 reference strain.

## ANNOUNCEMENT

Fructophilic lactic acid bacteria (FLAB) are a relatively recently described group of microorganisms that includes bacteria that prefer fructose and thus environments rich in this sugar, such as flowers, vegetables, and digestive tracts of insects (1–3). NADH oxidation pathways in FLAB lead to the conversion of fructose to mannitol, prompting researchers to look for new strains with the potential to produce this polyol (4–6).

We isolated Apilactobacillus kunkeei strain 7K4AA and A. kunkeei strain 7K11C from flowers (51°15′N, 22°30′E) according to methods used in previous studies (7). Strains were selected for identification after high-performance liquid chromatography (HPLC) screening against mannitol (from MRS medium [8] with 4% fructose, strain 7K4AA produced 0.49% mannitol after 24 h, strain 7K11C produced 1.17%, and reference strain DSMZ 12361 produced 0.14%) (9). Species identification was performed by 16S rRNA gene sequencing and matrix-assisted laser desorption ionization–time of flight mass spectrometry (MALDI-TOF MS) (7). The three strains of A. kunkeei were cultured at 30°C for 24 h in MRS medium with 2% fructose. Genomic DNA was extracted from single colonies using the Genomic Maxi AX kit with RNase (A&A Biotechnology, Poland). Briefly, a paired-end library was constructed by using the NEBNext Ultra DNA library preparation kit for Illumina (New England Biolabs, Ipswich, MA, USA) and subsequently sequenced on a MiSeq sequencer with 2 × 300-bp paired-end sequencing chemistry (Illumina, San Diego, CA, USA). Furthermore, the ligation sequencing kit 1D (SQK-LSK109) and native barcoding expansion 1–12 PCR-free kit were used to generate a 1D long-read library (Oxford Nanopore Technologies, Oxford, UK). Purified DNA was sequenced on a MinION sequencer (Oxford Nanopore Technologies) for 48 h with a SpotON flow cell MK I (R9.4.1). The base calling from Nanopore sequencing data was performed using Guppy v4.0.15 software (Oxford Nanopore Technologies). Read trimming and filtering were performed using Cutadapt v1.18 (10). *De novo* sequence data assembly from both sequencing platforms was carried out using Unicycler v0.4.7 software (11). Annotation was performed with the NCBI Prokaryotic Genome Annotation Pipeline (PGAP) (12). Default parameters were used for all software unless otherwise specified. The sequencing statistics are shown in Table 1.

**TABLE 1:** Sequencing statistics

Genome characteristic	Data for:		
	A. kunkeei strain 7K11C	A. kunkeei strain 7K4AA	A. kunkeei strain DSMZ 12361
Total length (bp)	163,444	159,735	154,222
GC content (%)	37.0	36.97	36.60
No. of CDSs	1,459	1,416	1,356
No. of tRNAs	78	64	64
No. of rRNAs	18	15	15
No. of other RNAs	3	3	3
No. of pseudogenes	2	3	3
No. of contigs	24	2	1
Total no. of reads	823,035	807,750	1,108,952
No. of MiSeq raw reads	631,078	705,389	944,158
Total size of MiSeq reads (bp)	189,323,400	211,616,700	283,247,400
MiSeq read *N*_50_ (bp)	300	300	300
No. of MinION raw reads	191,957	103,470	166,135
Total size of MinION reads (bp)	1,133,801,534	700,301,503	1,411,326,599
MinION read *N*_50_ (bp)	7,073	7,694	8,865
GenBank accession no.	JAIHBH000000000 (chromosome) JAIHBH010000003.1 (plasmid unnamed1) JAIHBH010000004.1 (plasmid unnamed2) JAIHBH010000006.1 (plasmid unnamed3)	CP080569.1 (chromosome) CP080570.1 (plasmid)	CP080568.1
BioSample accession no.	SAMN20804187	SAMN20557569	SAMN20557570
SRA accession no.			
MinION reads	SRX12680588	SRX12697028	SRX12697789
MiSeq reads	SRX12680587	SRX12697027	SRX12697788

The sequence length of the new strains exceeds the average for this species (1.55 Mb), with GC contents of 37% for strain 7K11C and 36.97% for 7K4AA. The highest number of coding sequences (CDSs) was detected in the genome of strain 7K11C, i.e., 1,459 CDs, with 43 fewer in 7K4AA and 1,356 in the reference genome. Numerous RNA coding sequences were discovered, as follows: for strain 7K11C, 78 tRNAs, 18 rRNAs, and 3 other RNAs; for strain 7K4AA, 64 tRNAs, 15 rRNAs, and 3 other RNAs; for strain DSMZ 12361, 64 tRNAs, 15 rRNAs, and 3 other RNAs. Strain 7K4AA has one plasmid of 31,379 bp (2.91× coverage), while three circular contigs (potential plasmid sequences) of 20,590 bp (0.44× coverage), 8,433 bp (28.73× coverage), and 6,522 bp (1.73× coverage) were found in the sequence of strain 7K11C. Plasmids, among other factors, add survival value to these microorganisms; therefore, a detailed analysis of both plasmid and chromosome sequences provides new insights into the unique lifestyle of FLAB (13, 14).

### Data availability.

The whole-genome shotgun projects have been deposited in NCBI GenBank. The BioSample, SRA, and GenBank accession numbers for A. kunkeei strain 7K11C, A. kunkeei strain 7K4AA, and A. kunkeei strain DSMZ 12361 are listed in Table 1.
